# Study of Muscle Cell Dedifferentiation after Skeletal Muscle Injury of Mice with a Cre-Lox System

**DOI:** 10.1371/journal.pone.0016699

**Published:** 2011-02-03

**Authors:** Xiaodong Mu, Hairong Peng, Haiying Pan, Johnny Huard, Yong Li

**Affiliations:** 1 The Laboratory of Molecular Pathology, Stem Cell Research Center (SCRC), Children's Hospital of UPMC, Pittsburgh, Pennsylvania, United States of America; 2 Department of Orthopaedic Surgery, University of Pittsburgh, Pittsburgh, Pennsylvania, United States of America; 3 Department of Bioengineering, University of Pittsburgh, Pittsburgh, Pennsylvania, United States of America; 4 Department of Pathology, University of Pittsburgh, Pittsburgh, Pennsylvania, United States of America; Ohio State University, United States of America

## Abstract

**Background:**

Dedifferentiation of muscle cells in the tissue of mammals has yet to be observed. One of the challenges facing the study of skeletal muscle cell dedifferentiation is the availability of a reliable model that can confidentially distinguish differentiated cell populations of myotubes and non-fused mononuclear cells, including stem cells that can coexist within the population of cells being studied.

**Methodology/Principal Findings:**

In the current study, we created a Cre/Lox-β-galactosidase system, which can specifically tag differentiated multinuclear myotubes and myotube-generated mononuclear cells based on the activation of the marker gene, β-galactosidase. By using this system in an adult mouse model, we found that β-galactosidase positive mononuclear cells were generated from β-galactosidase positive multinuclear myofibers upon muscle injury. We also demonstrated that these mononuclear cells can develop into a variety of different muscle cell lineages, i.e., myoblasts, satellite cells, and muscle derived stem cells.

**Conclusions/Significance:**

These novel findings demonstrated, for the first time, that cellular dedifferentiation of skeletal muscle cells actually occurs in mammalian skeletal muscle following traumatic injury *in vivo*.

## Introduction

The induction of multipotent or pluripotent stem cells from differentiated cells, known as dedifferentiation, has been intensively studied over the past number of years. Recently, a standard technique for the generation of induced pluripotent stem (iPS) cells from adult somatic cells has been established [1]; however, the cell sources for these stem cell induction studies are typically from adult tissues where adult stem cells are also known to exist [2], [3], [4]. Moreover, some documentation suggests that adult stem cells in some tissues are pluripotent [5], [6], [7]. Based on the heterogeneous nature of the various cell sources utilized, it has been suggested that some of the cell isolation techniques might not exclude all the stem cells from the isolation, leaving the possibility that it is actually the contaminating stem cells that are being induced and expanded in these studies rather than the dedifferentiation of “terminally” differentiated somatic cells [8]. From a cellular biology standpoint, it is very important to ensure that the cells that are actually undergoing transformation during cell dedifferentiation process are truly differentiated somatic cells and not undifferentiated stem or progenitor cells which can coexist in the tissue source [1], [8]. Therefore, a model that could provide and ensure that the cells utilized during the dedifferentiation procedure are actually terminally differentiated somatic cells would be very beneficial for these types of studies [8], [9].

It is generally believed that the dedifferentiation process of muscle cells occurs in injured skeletal muscle of some amphibians, but is still questioned to occur in mammalian muscle. Skeletal muscle is a good model for studying the process of dedifferentiation due to its high regenerative capacity and the fact that the terminally differentiated muscle cells are myofibers generated from myogenic fusion [10], [11]. In amphibian limb regeneration (i.e., newt and axolotl), it is the multipotent stem cells which are generated via the dedifferentiation of myofibers that are the major cell source for “blastema” formation and consequently “perfect” limb regeneration [11]. The multinuclear-to-mononuclear transition of muscle cells is a reversal of the myogenic fusion process and is an important and necessary step of myogenic dedifferentiation. Myogenic dedifferentiation in amphibians has been well documented, including the generation of stem cells from myofibers of injured amphibian [11], [12]. In mammalian systems, previous studies on the induction of muscle cell dedifferentiation have been claimed to be successfully conducted at two levels *in vitro* including: the dedifferentiation of myotubes/myofibers into myocytes and the dedifferentiation of myocytes into stem cell-like cells [13], [14]. However, this phenomenon still remains controversial. For example, in dedifferentiation studies involving myotubes, the myotubes formed by myocyte fusion could have been contaminated with non-fused myocytes and in the experiments involving the isolation of myofibers, the myofibers could certainly have possessed contaminating satellite cells or stem cells. Furthermore, a similar problem exists with the studies involving the dedifferentiation of myocytes into stem cells, due to the heterogeneous nature of the isolated primary cells because it is impossible to know, with absolution, that the cells were completely void of undifferentiated cells (i.e., satellite cells or stem cells) [15]. Therefore, it is reasonable to suggest the use of a method to specifically tag differentiated multinuclear myotubes and any mononuclear myocytes released from the multinuclear myotubes in order to confirm that the isolated cells are truly a credible source of differentiated cells for the aforementioned stem-cell induction studies.

The fusion of muscle cells to form multinuclear myofibers is central to muscle development and has been historically thought to be an irreversible process in mammals. Based on the cell-fusion characteristics of muscle cells, we have created a Cre/Lox-β-galactosidase (Cre-Lox) system [16] to specifically tag differentiated multinuclear myofibers, as well as mononuclear cells released from these tagged multinuclear myofibers via the *in vivo* dedifferentiation of the skeletal muscle after injury. We also isolated muscle cells from injured skeletal muscle tagged with the Cre-Lox system to further characterize the mononuclear cells generated and released from these myofibers in the injured muscle.

## Results

### Specificity of Cre-Lox system in tagging differentiated myotubes *in vitro*

**Figure 1:**
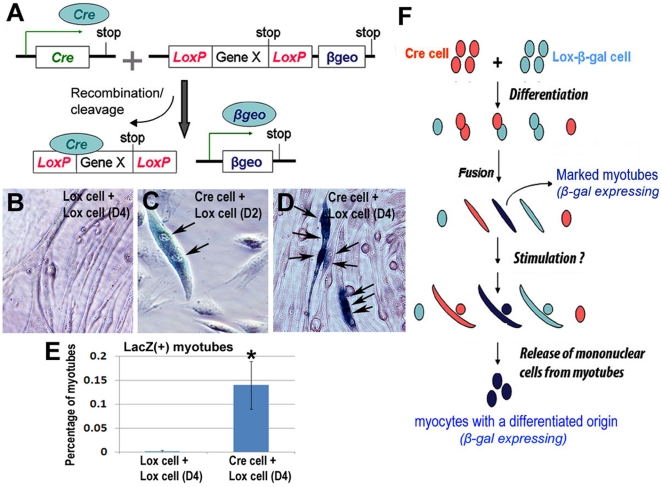
Specificity of the Cre-Lox system in differentiated myotubes *in vitro*. Schematic representation of the Cre/Lox-β-galactosidase system used in the current study (**A**). (β-gal/LacZ)+ cells were not observed with homogenous fusion of MDC-Lox cells (**B**). In co-cultured MDC-Cre and MDC-Lox cells, (β-gal/LacZ)+ cells can be either nascent myotubes with 2 nuclei (**C**, nuclei marked by arrows; 2 days after myogenic differentiation), or mature myotubes with multiple nuclei (**D**, nuclei marked by arrows; 4 days after myogenic differentiation). No (β-gal/LacZ)+ mononuclear cells were observed. Statistical quantification of (β-gal/LacZ)+ myotubes is shown (**E**). It is proposed that the presence of any mononuclear cells positive for β-gal expression in the culture have to be released from (β-gal/LacZ)+ myotubes, that is, after some type of stimulation, myotubes formed through the fusion of MDC-Cre and MDC-Lox cells, dedifferentiate and release the mononuclear (β-gal/LacZ)+ cells (**F**).

Muscle-derived cells (MDCs) from normal mice (C57BL6J, 4–6 weeks of age, male, Jackson Lab) were isolated and transfected with a Cre-expressing retroviral vector to generate MDC-Cre. The Cre gene (*Cre*, a DNA recombinase) is driven by a Muscle Creatine Kinase (*MCK*) promoter that is active in differentiating and differentiated muscle cells, such as activated myoblasts and myotubes [17]. Another population of MDCs were isolated from 129-Gt(ROSA)26Sortm1Sho (Lox-β-gal) reporter mice (MDC-Lox, 5–6 weeks of age, male, Jackson Lab). When MDC-Cre cells were co-cultured with MDC-Lox cells, the possible fusion of the two kinds of cells resulted in Cre recombinase-mediated cleavage of Lox sites and the induction of β-gal/LacZ expression in the fused/differentiated myotubes (Fig. 1A). To test the specificity of this Cre-Lox system *in vitro*, MDC-Cre and MDC-Lox cells were mixed (1∶1) in differentiation medium to promote muscle cell fusion. Homogeneous fusion of MDC-Lox cells were also studied as a control group to exclude the possibility that the Lox-β-gal reporter may express without Cre-mediated DNA recombination (Fig. 1B). The result showed that the β-gal/LacZ signal was not observed in the homogenous MDC-Lox cells, but was exclusively observed in multinuclear myotubes in the co-culture of MDC-Cre and MDC-Lox cells (Fig. 1B–E). This observation demonstrates the specificity of our Cre-Lox system for tagging terminally differentiated muscle cells. This finding also suggests that if any β-gal/LacZ positive mononuclear cells exist in the culture, they must have been generated and released from the differentiated β-gal/LacZ positive multinuclear myotubes, and can therefore be considered as dedifferentiated myocytes having an origin from differentiated cells/tissue (Fig. 1F).

### Transplantation of cells transfected with the Cre-Lox system into mice and observation of β-gal/LacZ positive cells in injured muscle

**Figure 2:**
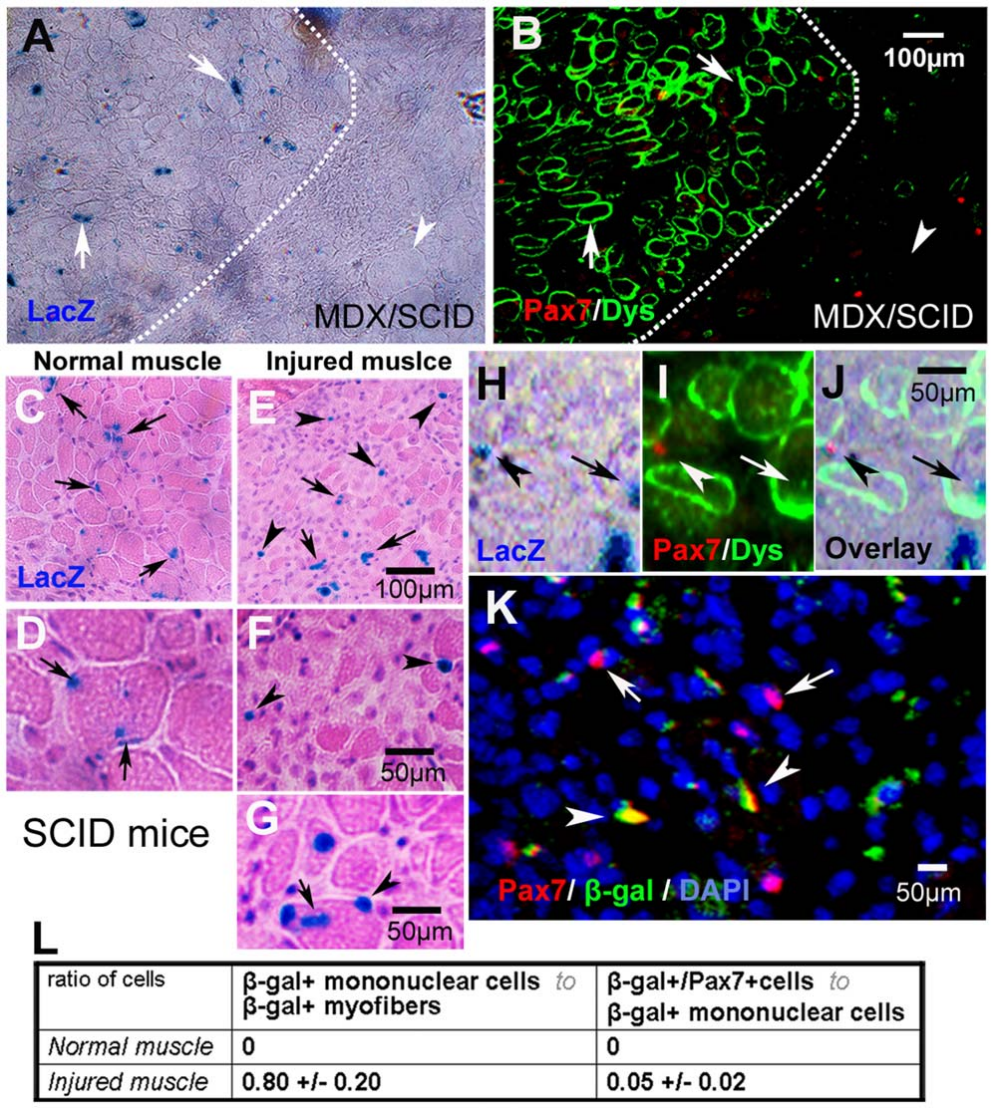
Cre/Lox-β-galactosidase cell transplantation and muscle injury resulted in β-gal/LacZ positive mononuclear cells. Three weeks after cell transplantation into MDX/SCID mice, many (β-gal/LacZ)+/dystrophin+ myofibers were observed (**A**, **B**). β-gal/LacZ staining and immunofluorescent Pax7/dystrophin staining are shown here; arrows indicate (β-gal/LacZ)+/dystrophin+ myofibers which are the result of the fusion of Cre-Lox myocytes, and arrowheads indicate (β-gal/LacZ)−/dystrophin− host myofiber (**A**, **B**). Three weeks after transplantation and four days after muscle injury in SCID mice, some (β-gal/LacZ)+ mononuclear cells were observed in the injured muscle (**E–G**), but not in the control non-injured muscle (**C**, **D**). Results of β-gal/LacZ staining and HE staining are shown here; arrows indicate (β-gal/LacZ)+ myofibers, and arrowheads indicate (β-gal/LacZ)+ mononuclear cells (**C–G**). Some of the (β-gal/LacZ)+ mononuclear cells were shown to be Pax7+ (**H–K**). Images H–J are of the same location, and result of β-gal/LacZ staining and immunofluorescent Pax7/dystrophin staining are shown here (**H–J**); arrows indicate β-gal+/Pax7+ mononuclear cells, and arrowheads indicate β-gal+ myofibers (**H–J**). Fluorescent co-staining of β-gal and Pax7 are also shown (K); arrowheads indicate β-gal+/Pax7+ mononuclear cells, and arrows indicate Pax7+ cells. Statistical quantification of the results is also shown (**L**).

After creating the model and verifying its specificity *in vitro*, we utilized the system *in vivo* to investigate whether mononuclear myocytes could be generated from differentiated myofibers and whether these dedifferentiated myocytes could then be further dedifferentiated into SC-like cells in the injured muscle of mice. Firstly, to verify the efficiency of the Cre-Lox system *in vivo*, equal numbers of MDC-Cre and MDC-Lox cells (0.3×10^6^ cells each) were mixed and implanted via intramuscular injection into the gastrocnemius (GM) muscles of MDX/SCID mice, an immuno-deficient, dystrophic mouse model, and allowed to completely differentiate and fuse within the host muscle. Three weeks after implantation, many β-gal/LacZ positive myofibers were observed and no obvious β-gal/LacZ positive mononuclear cells were detected at the cell implantation site (Fig. 2A&B), verifying that the Cre-Lox system also functioned *in vivo*. In the next set of experiments, we implanted the same MDC-Cre and MDC-Lox cell mix that we utilized above into the non-injured muscle of SCID mice. Three weeks after cell implantation, we observed similar results that we had in the MDX/SCID mice (Fig. 2C&D); however, when a laceration injury was created in the GMs of these mice at the cell implantation site, mononuclear cells that were β-gal/LacZ positive were detected four days after the creation of the injury (Fig. 2E–G, arrowheads). Moreover, a sub-population of the β-gal/LacZ positive mononuclear cells that was also positive for Pax7 was observed in the injured muscles (Fig. 2H–K, arrowheads). Pax7 is a cell marker of satellite cells that is no longer expressed when the cells are committed to differentiate or have already differentiated. These latter results also suggest that the population of β-gal/LacZ positive mononuclear cells may be heterogeneous and contain different subpopulations. These results demonstrated that differentiated myofibers (β-gal/LacZ positive fibers) could dedifferentiate into Pax-7 expressing precursor cells after myofiber injury, which indicates that dedifferentiation may play a role in the healing process of mammalian skeletal muscle.

### β-gal/LacZ positive mononuclear cells are present in different cell populations from the preplate isolated muscle cells

**Figure 3:**
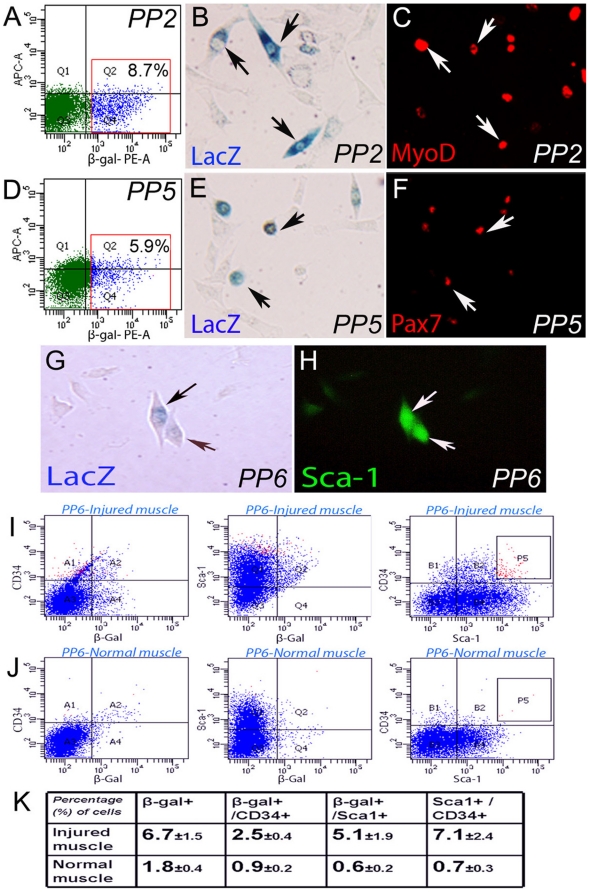
β-gal/LacZ positive mononuclear cells from injured muscle contain different cell populations (i.e., myoblasts, satellite cells, and MDSCs). The result of flow cytometry analysis, β-gal/LacZ staining and immune-staining to MyoD or Pax7 showed that, the isolated PP2 (myoblasts) (**A–C**) and PP5 (satellite cells) (**D–F**) cell populations both contain (β-gal/LacZ)+ cells, which can be MyoD+ or Pax7+. The result of β-gal/LacZ staining and immune-staining to Sca-1 also showed that, the PP6 cells (MDSCs) also contain (β-gal/LacZ)+ cells, which were shown to be Sca-1+ (**G**, **H**). Flow cytometry analysis comparing PP6 cells from non-injured control muscles and the PP6 cells from injured muscle demonstrated a greater number of (β-gal/LacZ)+ cells which also expressed Sca-1 and CD34 in the injured muscle (I–J), indicating that adult stem cells can be dedifferentiated from “terminally” differentiated muscle fibers in injured skeletal muscle of mice. Statistical quantification of the flow cytometry results is shown (**K**).

In order to further confirm the generation of dedifferentiated β-gal/LacZ positive mononuclear cells in the injured muscle, and in order to find out what cell populations contain these β-gal/LacZ positive cells, we isolated muscle cells from the injured and non-injured skeletal muscles of SCID mice that contained β-gal/LacZ positive multinuclear myofibers and mononuclear cells 4 days after muscle injury. With flow cytometry, we analyzed the population for its percentage of β-gal/LacZ positive mononuclear cells among the total number of isolated mononuclear cells. Different populations of cells can be separated using the pre-plate technique which separates cells based on their different affinities for adhering to collagen-coated flasks [18], [19]. For example, myoblasts adhere early on, usually after the 2^nd^ or 3^rd^ preplate (i.e. PP2 or PP3) passage, satellite cells adhere at approximately PP5, and muscle derived stem cells adhere at PP6. The cells isolated from the injured skeletal muscles of SCID mice showed that β-gal/LacZ positive mononuclear cells exist in both populations of myoblasts (Fig. 3A–C) and satellite cells (Fig. 3D–F), as well as in the Muscle Derived Stem Cell (MDSC) population (Fig. 3G–H), indicating that β-gal/LacZ positive myocytes should be able to take part in the re-construction of injured muscle. More importantly, many more β-gal/LacZ positive cells expressing the SC markers Sca-1 and CD34 [18] were observed in the population of cells isolated from injured muscle, compared to normal non-injured muscle (Fig. 3I–K). These results verify the existence of different populations of β-gal/LacZ positive mononuclear cells in the healing process, and suggest that dedifferentiation can be induced via muscle injury and that muscle progenitor cells (i.e., satellite cells and MDSCs) can be generated through the process of dedifferentiation. This observation suggests that the increased population of stem cells present in injured muscle tissue is comprised of not only satellite cells, stem cells, and circulating blood cells, but also by cells that are being generated through the process of dedifferentiation from “terminally” differentiated myofibers.

### β-gal/LacZ positive mononuclear cells can proliferate, and contribute to myotube formation *in vitro*, as well as contribute to re-vascularization in injured skeletal muscle *in vivo*

**Figure 4:**
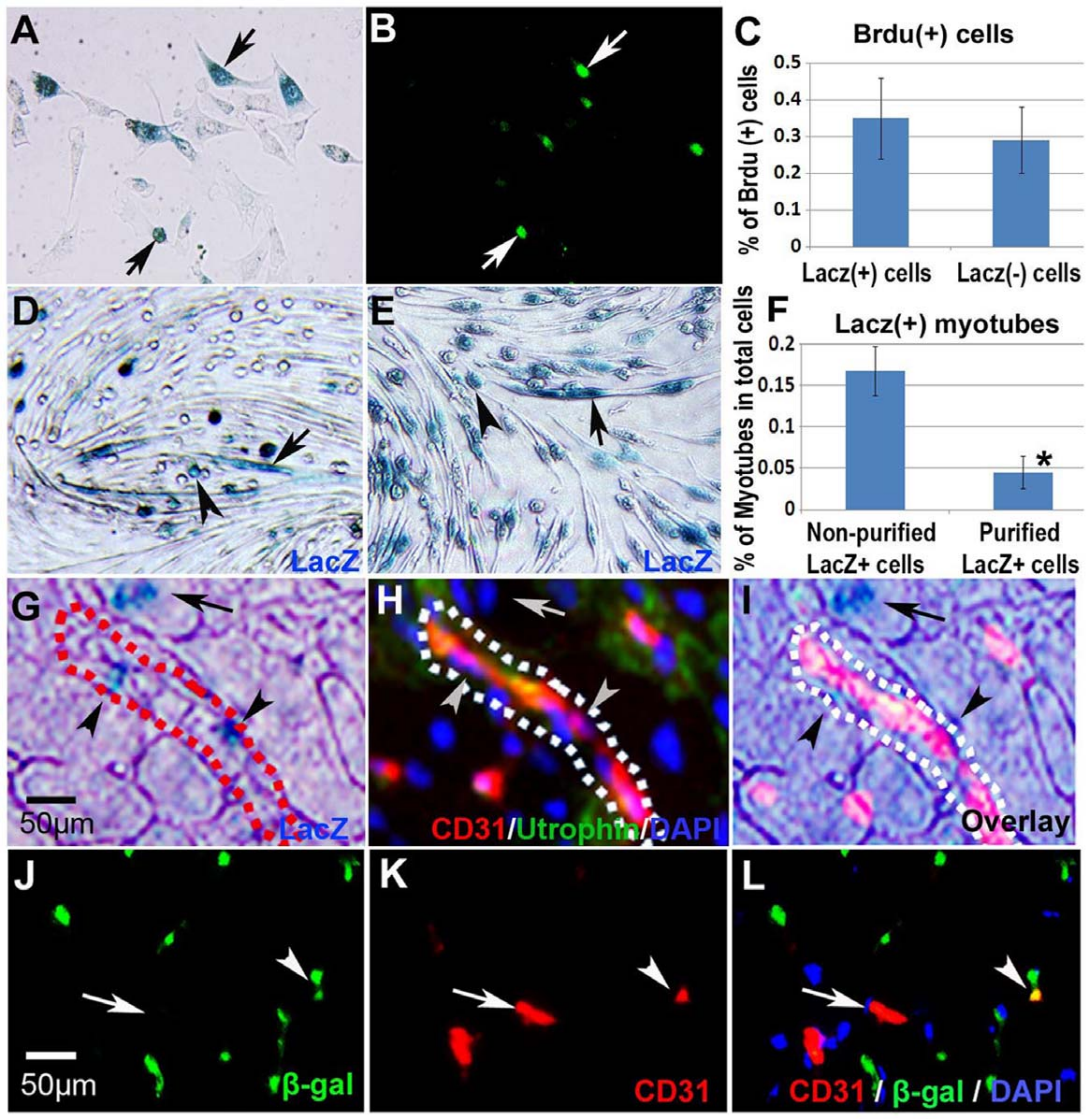
β-gal/LacZ positive cells can proliferate and contribute to myotube formation *in vitro*, and blood vessel formation *in vivo*. BrdU incorporation assay showed that (β-gal/LacZ)+ cells can proliferate (**A–C**). A myogenic differentiation assay, which deprives the cultures of serum, showed that (β-gal/LacZ)+ cells, in both cell populations without purification [ around 6% of cells were (β-gal/LacZ)+] (**D**) and after purification with Fluorescence-activated cell sorting (**E**), can participate in myotube formation (**F**). *In vivo*, ten days after laceration-injury of GM muscles that were transplanted with Cre-cells and Lox-cells for 3 weeks, some (β-gal/LacZ)+ signal was also found to co-localize with CD31+ signal in the blood vasculature (**G–L**). Images G–I or J–L are of the same location in tissue, and result of β-gal/LacZ staining and immunofluorescent CD31/Utrophin staining are shown here (**G–I**); arrowheads indicate β-gal+/CD31+ cells, and arrows indicate β-gal+ myofibers (**G–I**). Fluorescent co-staining of β-gal and Pax7 are also shown (**J–L**); arrowheads indicate β-gal+/Pax7+ cells, and arrows indicate CD31+ cells (**J–L**).

We continued to investigate whether the β-gal/LacZ positive mononuclear cells isolated via the preplate technique from the injured muscle of SCID mice injected with the Cre-Lox cells had the same proliferation and myogenic differentiation capacities typically observed by muscle progenitor cells. BrdU incorporation studies showed that β-gal/LacZ positive mononuclear cells could enter the S-phase of the cell cycle, indicating that the proliferation capacity of the cells is positive (Fig. 4A–C). The *in vitro* myogenic studies showed that β-gal/LacZ positive mononuclear cells (non-purified PP6 cells or purified β-gal/LacZ positive cells from PP6) were able to participate in myotube formation, although the purified β-gal/LacZ positive cells showed a slower myogenic differentiation process (Fig. 4E–F). Also, we noticed that the *in vivo* implantation of the Cre-Lox cell mix into the skeletal muscle of SCID mice resulted in the appearance of β-gal positive signal in both the myofibers and CD31 positive blood vasculature 10 days after muscle injury (Fig. 4G–L). This finding indicates that the β-gal/LacZ positive mononuclear cells released from myofibers after injury could differentiate into endothelial cells and participate in the re-vascularization of the tissue.

## Discussion

In this study, we have utilized a Cre/Lox-β-galactosidase (Cre-Lox) system [16], [20] to specifically tag multinuclear myofibers as well as any mononuclear cells released from the tagged myofibers via dedifferentiation *in vitro* and *in vivo*. Utilizing this Cre-Lox model, we not only created a system to identify truly differentiated muscle cells which will be available for further cell dedifferentiation studies, but were also able to demonstrate that dedifferentiation does occur in mammalian skeletal muscle following injury. We were able to show two tiers to the dedifferentiation including the dedifferentiation of myofibers to myocytes and the dedifferentiation of myocytes to even earlier progenitor cells including muscle-derived stem cells (CD34/Sca1 expressing cells). We also showed that these dedifferentiated cells were able to proliferate and undergo myogenic differentiation the same as any other muscle progenitor cell could do. In addition, the dedifferentiated cells were capable of differentiating into non-muscle cell related cell lineages (i.e., endothelial cells involved in revascularization of the injured tissue). Our results suggest that, in addition to resident stem cells and circulating stem cells, dedifferentiated muscle cells also appear to be a source of muscle progenitor cells contributing to the muscle healing process.

A variety of Cre-Lox models have been used in such a manner as to be explicitly activated in specific tissue types or cell types or only when the expression of certain gene(s) are modified in order to trace cell lineages [21], [22]. A Cre/Lox-β-galactosidase system, similar to ours, had been used to detect cell fusion events of bone-marrow-derived cells (BMDCs), neurons, and cardiomyocytes [23]. The Cre-Lox system was proven effective for specifically tagging differentiated myofibers to study the process of trans-differentiation in the development and tail regeneration of Xenopus, a genus of aquatic frogs [24]. In this novel study we used the Cre-Lox system to study the process of dedifferentiation in mammalian skeletal muscle. The results generated from these studies indicated that the Cre-Lox system can serve as an efficient tool for the study of muscle cell dedifferentiation and can be used to ensure that the somatic cells targeted in stem cell induction studies are truly terminally differentiated.

Potential problems with the Cre-Lox system when performing the dedifferentiation-differentiation studies may include the potential for spontaneous fusion of the Cre and Lox cells to form tetraploid hybrids without actually undergoing myogenic differentiation [25], [26], as well as the potential secretion and lateral transfer of the Cre protein from the Cre-cells to the Lox-cells [27]. In both of these two hypothetical situations, β-gal expression could occur in non-differentiated mononuclear cells; however, the spontaneous fusion of skeletal muscle myoblasts to form tetraploid hybrids has never been documented. Moreover, the Cre gene in our system is driven by an MCK promoter which is only active in differentiating and mature differentiated muscle cells [17], which minimizes the possibility of Cre expression in non-differentiated mononuclear cells. Furthermore, in our experiments, no β-gal positive mononuclear cells or non-differentiated cells were observed during the *in vitro* co-culture and myogenic differentiation of the Cre-cells and Lox-cells. This observation suggests that the possibility of spontaneous fusion or lateral transfer of the Cre protein, if any, would be limited, and would not contribute significantly to the generation of β-gal positive mononuclear cells. In fact, a small number of β-gal/LacZ positive mononuclear cells was also observed in non-injured normal muscle implanted with Cre-Lox cells in our study, which could be related with the minor injuries in the normal muscle induced by strenuous exercise or unaccustomed movement of the animals [28].

In conclusion, we have created a Cre-Lox model to study the process of myogenic dedifferentiation and a method to obtain confirmed “terminally” differentiated myogenic cells for use in dedifferentiation studies. In addition, this research led to the confirmation for the first time that dedifferentiation does indeed occur in the injured skeletal muscle of mice, and that the dedifferentiation of “terminally” differentiated cells may constitute an additional cell source to aid in the healing process of injured skeletal muscle. Our results provide a greater understanding of the skeletal muscle healing process after injury or from disease, and will shed light on the role of cellular dedifferentiation in regenerative medicine. Additional studies are needed to confirm this similar phenomenon in other mammalian species.

## Materials and Methods

### Cre-Lox system

The use of animals and the surgical procedures performed in this study were approved by the Institutional Animal Care and Use Committee (IACUC) of University of Pittsburgh Medical Center (Protocol 0904641). Muscle-derived cells (MDCs) were isolated from normal mice (C57BL/6J, 4–6 weeks of age, male, Jackson Laboratory) and transfected with a Cre-expressing retroviral vector to make MDC-Cre. The Cre-retroviral vector was built with a pCLXSN retroviral expression vector (Addgene) which is under the control of the MCK promoter and also carries a neomycin-resistance gene (**Fig. 1A**). MDCs transfected with the Cre-retroviral vector were selected with G418 (1 mg/mL) for 2 passages over a 5 day period to obtain a purified Cre-expressing cell population. MDC-Lox were isolated from Lox-β-gal reporter mice (B6;129-Gt(ROSA)26Sor^tm1Sho^/J, 5–6weeks of age, male, Jackson laboratory), in which expression of the β-gal reporter gene is silenced by a LoxP-flanked stop codon. Fusion of MDC-Cre and MDC-Lox cells resulted in Cre-mediated cleavage of the Lox sites and the induction of β-galactosidase (β-gal) expression, which is regulated with a CMV promoter. This Cre-Lox system effectively tags differentiated myotubes and myofibers and enables us to track their fate both *in vitro* and *in vivo*.

### Cell transplantation of MDCs to the skeletal muscle of mice

All experimental animal protocols were approved by the Animal Research and Care Committee in the Children's Hospital of Pittsburgh of UPMC. The mice utilized in these studies were either SCID mice (C57BL/6J-*Prkdc^scid^*/SzJ, female, 4-week old, Jackson Laboratory) or MDX/SCID mice (bred in the institution's animal facility by crossing C57BL/10ScSn-*Dmd^mdx^*, male, Jackson Laboratory, and C57BL/6J-*Prkdc^scid^/*SzJ mice, female, 4-week old, Jackson Laboratory). For all experiments involving cell transplantation with the Cre/Lox system, equal numbers of MDC-Cre and MDC-Lox (0.3×10^6^ cells each) were mixed and transplanted into the gastrocnemius (GM) muscles in both legs of mice for up to 3 weeks, allowing for fusion of the transplanted cells into myofibers before creating the laceration muscle injury.

### Skeletal muscle injury

GM muscles of mice that had been transplanted with MDC-Cre/MDC-Lox 3 weeks earlier were then injured via a laceration at the site of the implanted cells. Lacerations were performed on the GM muscle of one leg of each mouse through 50% of its width and 100% of its thickness at 60% of its length, which covered the location of the implanted cells. The GM muscle of the other leg served as a non-injured control.

### Isolation of skeletal muscle cells

Four days after muscle laceration injury the mice were sacrificed, and the skeletal muscle cells were isolated from the GM muscle of the mice. Using a modified preplate technique [18], [19], different populations of muscle cells were isolated and cultured on collagen-coated flasks. The harvested GM muscle was cut and minced into a coarse slurry. The muscle slurry was then digested by serial 1-hr incubations at 37°C in 0.2% type XI collagenase (Sigma), dispase (grade II, 240 units; Sigma), and 0.1% trypsin (GIBCO-BRL). The cell suspension was then passed through 22-gauge needles, centrifuged at 3000 rpm for 5 min, and resuspended in growth medium (Dulbecco's modified Eagle's medium supplemented with 10% Fetal Bovine Serum (FBS), 10% Horse Serum (HS), 1% Penicillin-Streptomycin antibiotics, and 0.5% chicken embryo extract (CEE)]; all from GIBCOBRL). After the cells were plated in collagen-coated flasks as indicated by the pre-plate technique, the fast adhering cells (i.e., PP1 and PP2) were then cultured with growth medium for myoblasts as outlined above, and the slow adhering cells (i.e., PP5 and PP6) continued to be cultured in growth medium to isolate and expand the stem cells (The same component as medium for myoblasts except with 20% FBS), and incubated in 5% CO_2_ at 37°C.

### β-gal/LacZ staining and immunofluorescent staining of cells and tissue sections

GM muscle tissues from SCID or MDX/SCID mice were harvested and snap frozen in liquid nitrogen-cooled 2-methylbutane and cryo-sectioned at 10µm. Cultured cells were fixed with 4% paraformaldehyde and tissue cryo-sections were fixed with 4% formalin. For β-gal/LacZ staining, fixed cells/sections were incubated in 5-bromo-4-chloro-3-indolyl-β-d-galactoside (X-gal) solution at 37°C for 24 hours. For immunofluorescent staining, 10% HS was used to block nonspecific background for 1 hour. The primary antibodies, Pax7 (DHSB), Dystrophin (Abcam), MyoD (Santa Cruz), CD34 (BD Biosciences), Sca-1 (BD Biosciences) were applied at 1∶100∼200. After incubating with fluorescent secondary antibodies, fluorescence microscopy (Leica Microsystemic Inc., IL) was used to visualize all of the immunofluorescence results and capture photographic images.

### Flow cytometry study of isolated muscle stem cells

PP6 cell populations from our MDX/SCID mice or SCID mice were characterized by flow cytometry for CD34 and Sca-1 expression. PP6 cells were labeled with biotin-conjugated anti–mouse Sca-1 and CD34 Abs (BD Biosciences). A separate portion of cells was treated with equivalent amounts of isotype control antibodies. Both fractions were washed and labeled with fluorescent-tagged streptavidin-allophycocyanin. Sca-1 and CD34 expression was determined by flow cytometry with a cell sorter (FACStar Plus or FACSAria; Becton Dickinson).

### Bromodeoxyuridine (BrdU) incorporation assay

Muscle cells were isolated from the injured muscle of our SCID mice and cultured with BrdU (BD Bioscience) (1∶1000) in proliferation medium for 16 hrs, as described previously [29]. The cells were then fixed with 4% paraformaldehyde and immunostained with an antibody against BrdU (Abcam). β-gal/LacZ staining was then performed and the presence of cells colocalized with BrdU (cells which are undergoing proliferation) and β-gal/LacZ (dedifferentiated cells) were determined. The percentages of BrdU positive mononuclear cells in the populations of β-gal/LacZ positive and β-gal/LacZ negative mononuclear cells were then quantified.

### Flow cytometric cell sorting of β-gal positive cells

Around 2×10^4^ muscle cells isolated from the injured muscles of our SCID mice were immunostained with an antibody against β-gal (sigma), and β-gal positive cells was determined and isolated by flow cytometry with a cell sorter (FACStar Plus or FACSAria; Becton Dickinson).The sorted cells were then immediately cultured in the proliferation medium.

### *In vitro* myogenic differentiation assay of β-gal/LacZ positive cells

A myogenic differentiation assay was conducted on both non-purified PP6 cells containing β-gal/LacZ positive cells and β-gal/LacZ positive cells purified from the PP6 cells by flow cytometric cell sorting, as described previously [30]. Briefly, cells were cultured in proliferation medium (PM) (DMEM supplemented with 10% HS, 10% FBS and 1% P/S) to 85% confluence. PM was then changed to myogenic differentiation medium (DMEM supplemented with 2% HS and 1% P/S), and incubated in 5% CO_2_ at 37°C for 4 days. Myotube formation was then quantitated.

### Statistical analysis

The *in vitro* and *in vivo* measurement and quantification of the mononuclear cells positive for BrdU or β-gal/LacZ and myotubes/myofibers positive for β-gal/LacZ was conducted using Northern Eclipse software (version 6.0, Empix Imaging Inc., Mississauga, ON, Canada). At least 3 identically treated cell cultures or tissue sections from 4 identically treated mice for each group were used to generate the data, which was analyzed based on 3 pictures of each cell culture or tissue section. All of the results from these studies are expressed as the mean ± standard deviation (SD). The differences between means were considered statistically significant if *P value* was <0.05. The ANOVA test was used to compare the differences between different groups of cells or tissue sections.
